# Hierarchical PANI/NiCo-LDH Core-Shell Composite Networks on Carbon Cloth for High Performance Asymmetric Supercapacitor

**DOI:** 10.3390/nano9040527

**Published:** 2019-04-03

**Authors:** Xinjin Ge, Ying He, Tomas Plachy, Natalia Kazantseva, Petr Saha, Qilin Cheng

**Affiliations:** 1 Key Laboratory for Ultrafine Materials of Ministry of Education, School of Materials Science and Engineering, East China University of Science and Technology, Shanghai 200237, China; 18818217542@163.com; 2 Centre of Polymer Systems, Tomas Bata University in Zlin, nam. Masaryka T.G. 5555, 760 01 Zlin, Czech Republic; plachy@utb.cz (T.P.); kazantseva@utb.cz (N.K.); saha@utb.cz (P.S.)

**Keywords:** polyaniline nanofibers, layered double hydroxides, core-shell structure, electrochemical performance, supercapacitor

## Abstract

In this work, a facile two-step strategy is adopted to construct hierarchical polyaniline/NiCo-layered double hydroxide (PANI/NiCo-LDH) core-shell composite nanofiber networks on carbon cloth (CC). Three-dimensional (3D) porous PANI nanofiber networks are firstly uniformly anchored on CC by in-situ oxidative polymerization, followed by growth of NiCo-LDH nanoflakes on the crosslinked PANI framework via electrochemical deposition. The morphology and electrochemical properties of PANI/NiCo-LDH composites are controlled by the deposition time of LDH. Benefiting from rapid electron transport and ion diffusion, the well-defined PANI/NiCo-LDH hierarchical composite with 200 s deposition of LDH delivers a large capacitance of 1845 F g^−1^ at 0.5 A g^−1^ and excellent cycling stability of 82% capacitance retention after 5000 cycles at a very high current density of 10.0 A g^−1^. Furthermore, an asymmetric supercapacitor (ASC) assembled with PANI/NiCo-LDH as a positive electrode and activated carbon (AC) as a negative electrode exhibits a high capacitance of 147.2 F g^−1^ in a potential range from 0 to 1.5 V and superior energy density of 46.0 Wh kg^−1^ at a power density of 351.6 W kg^−1^.

## 1. Introduction

With the ever-growing concern of energy shortage and environmental pollution, the exploitation of renewable, clean energy and efficient energy storage systems has become extremely imperative. As one of most promising charge storage devices, supercapacitors (SCs), consisting of electric double-layer capacitors and pseudocapacitors, have been one of the hotspots in current research due to their high power density, fast charge–discharge rate, long cycle life and high safety [1,2,3,4]. It has been demonstrated that the performance of SCs mainly depends on the kinetic characteristics and electrochemical activity of the electrodes [5]. Therefore, it is of scientific and engineering significance to develop novel electrode materials with more electroactive sites, fast electron transfer rate and short ion-diffusion paths. So far, various active materials including carbons [6,7], conducting polymers [8,9] and transition metal oxides [10,11,12,13,14] with unique architectures have been explored as electrode materials. Although considerable progress has been made in the SC’s area, it is still an important but pressing task to enhance their energy density, power density and cycling life simultaneously by rationally engineering electrodes with integrated nanoarchitecture.

Recently, transition metal oxides/hydroxides, especially NiCo-LDHs, have received much more attention as SC electrodes due to their highly specific capacitance, superior redox activity, low cost and being environmental benign [15,16,17,18,19]. Although NiCo-LDHs exhibit better electrochemical performance than the corresponding single hydroxides, they cannot meet the increasing energy demands for new energy storage systems because of their intrinsic properties such as low electrical conductivity, slow ion transfer rate and easy aggregation of active species [20,21]. An efficient strategy to overcome these drawbacks is to incorporate pseudocapacitive materials into LDH to form core-shell nanostructures [22,23,24,25], which not only greatly facilitate electrons transport and ion transfer, but also significantly accommodate the strain within the electrodes induced by the electrochemical process. As a result, the desirable core-shell nanoarchitecture endows NiCo-LDHs electrodes with enhanced capacitance and rate capability. However, the insufficient conductivity and structural stability still restrict the development of high-performance SCs.

Polyaniline (PANI), another kind of pseudocapacitive material, has also attracted intense interest over the past years owing to its high conductivity, excellent chemical stability and reversible redox behavior [26,27]. Unfortunately, unsatisfactory cycle life and poor mechanical properties upon the charge–discharge process limit its application in SCs. The combination with metal oxides into well-defined binary or ternary nanocomposites could be an effective way to eliminate the disadvantages and thus achieve high electrochemical performance. Until now, most reported PANI/metal oxide composites in which PANI serves as a shell show some structural defects [28,29]. The PANI shell often prevents the close contact between the metal oxides and electrolytes, giving rise to low electrochemical utilization of metal oxides; on the other hand, PANI structural instability still remains at high current density in spite of the improved specific capacitance of composites. Therefore, it is highly essential to develop an effective method for the fabrication of elaborate core-shell nanostructures in which all the active components involved in faradic reactions and fast charge/mass transport can be realized.

To address the above-mentioned issues, herein we report a well-connected hierarchical composite structure consisting of a conductive PANI nanofibers core and NiCo-LDH nanosheets shell via in-situ polymerization and electrodeposition method. The mutual crosslinked conductive PANI nanofibers constitute a 3D network, providing sufficient surface area for the deposition of NiCo-LDHs. The porous networks give electrolyte ions full access into active materials leading to more redox reactions. Meanwhile, abundant pores in the composite networks serve as electrolyte reservoirs to shorten electrons and electrolyte ions transport distance. We believe that the synergistic effect derived from LDH shell and PANI core also boosts the overall electrochemical behavior of the composite. Hence, the as-synthesized core-shell nanostructure exhibits outstanding electrochemical performance. At a current density of 0.5 A g^−1^, PANI/NiCo-LDH possesses a highly specific capacitance of 1845 F g^−1^ and good rate capability with 82.1% capacitance retention from 1 A g^−1^ to 10 A g^−1^. Moreover, the fabricated asymmetric supercapacitor (ASC) exhibits a high energy density of 46.0 Wh kg^−1^ at a power density of 351.6 W kg^−1^ and impressive specific capacitance of 147.2 F g^−1^. 

## 2. Experimental

### 2.1. Materials

Carbon cloth (CC, BET surface area is around 27 m^2^ g^−1^) was commercially available from CeTech Co., Ltd. (Taiwan, China). Nickel nitrate hexahydrate (Ni(NO_3_)_2_·6H_2_O) and cobalt chloride hexahydrate (CoCl_2_·6H_2_O) were purchased from Sigma-Aldrich Company (St. Louis, MO, USA). Aniline (AN, 99.5%), phytic acid (50%) and activated carbon (AC, 99.9%) were obtained from Macklin Biochemical Co., Ltd. (Shanghai, China). All reagents were used without further purification.

### 2.2. Synthesis of PANI Nanofiber Network

PANI nanofibers were synthesized on carbon cloth (CC) via in-situ polymerization similar to a previous report [30]. Prior to the experiment, a piece of CC (1 × 2 cm^2^) was successively pretreated with aqua regia, ethanol and deionized water to eliminate impurities. In a typical synthetic process, 0.1 mg (0.43 mmol) ammonium persulfate (APS) was dispersed into 0.6 mL deionized water to form solution A. Then solution B was prepared by mixing 55 µL (0.6 mmol) aniline, 82 µL (0.09 mmol) phytic acid and 1 mL deionized water. Solution A and solution B were put into the refrigerator and cooled to 0–4 °C. Then a piece of CC was immersed in solution B and mixed with solution A quickly. The polymerization was carried out at 0–4 °C for 8 h. After the reaction, the CC with green PANI was cleaned with deionized water and absolute ethyl alcohol several times and then dried in a vacuum oven at 60 °C for 12 h. 

### 2.3. Preparation of Hierarchical PANI/LDH Core-Shell Nanostructures

Hierarchical PANI/LDH core-shell nanocomposites were synthesized via a facile electrochemical deposition method in a three-electrode system. The resulting PANI on CC was used as a working electrode, and the Ag/AgCl electrode and platinum sheet were applied to the reference electrode and counter electrode, respectively. LDH was coated on the surface of PANI by electrodeposition in a mixed solution containing 0.15 M Ni(NO_3_)_2_·6H_2_O and 0.15 M CoCl_2_·6H_2_O. with a constant potential of −1.0 V for 50–250 s at room temperature; the obtained samples were designated as PANI/LDH-T (T = 50, 100, 150, 200, and 250 s); and the corresponding mass loading of electrodes are 1.1, 1.4, 1.9, 2.5, and 3.3 mg cm^−2^, respectively. Finally, the product was rinsed with distilled water and ethanol several times and then dried in an oven for 12 h. In addition, pure PANI and NiCo-LDH growing on CC were named as PANI/CC and LDH/CC, respectively.

Furthermore, the weight percent of NiCo-LDH in composites is calculated to be 1.6, 3.9, 7.5, 11.5 and 16.3% for PANI/LDH-T (T = 50, 100, 150, 200, 250 s) by the weight difference before and after synthesizing PANI/LDH on CC using a high-precision balance.

### 2.4. Material Characterization 

The crystallization structures of samples were measured by X-Ray diffraction (XRD, Rigaku Corporation, Tokyo, Japan) equipped with Cu Kα radiation (λ = 0.15406 nm) under the accelerating voltage of 40 kV. The structure, morphology and compositions were further analyzed by field-emission scanning electron microscope (FESEM, S4800, HITACHI, Tokyo, Japan) with energy dispersive X-ray spectroscopy (SEM-EDX, Tokyo, Japan) and transmission electron microscope (TEM, JEM-2100F, JEOL, Tokyo, Japan). X-ray photoelectron spectroscopy (XPS, Thermo Fisher Scientific, Waltham, MA, USA) spectra were performed on an AXIS Ultra DLD spectrometer using a monochromatized Al Ka X-ray source (1486.71 eV). Fourier transform infrared (FTIR) spectra were recorded on a Nicolet 6700 FTIR spectrometer (Thermo Fisher Scientific, Waltham, MA, USA). Nitrogen adsorption–desorption isotherms of the samples were measured at liquid N_2_ temperature (77 K) using a JW-BK112T instrument (JWGB. SCI.&TECH Co., Beijing, China).

### 2.5. Electrochemical Measurements

The electrochemical properties of the samples were measured by a typical three-electrode system in 2.0 M KOH aqueous electrolyte at room temperature on a CHI-660E (Shanghai Chenhua Instrument Co., Shanghai, China) electrochemical workstation. Ag/AgCl electrode and platinum sheet (2 × 2 cm^2^) were selected as the reference electrode and counter electrode, respectively. Cycle voltammetry (CV) measurements were carried out in the potential range of −0.1∼0.5 V at various scan rates (5, 10, 20, 50, 100 mV s^−1^). The galvanostatic charge/discharge (GCD) was tested at different current densities of 0.5–10 A g^−1^ with a potential window ranging from 0 to 0.45 V. Electrochemical impedance spectroscopy (EIS) was carried out with a frequency range of 10^−2^–10^5^ Hz at open circuit potential. The specific capacitance of the composite electrode was calculated by the following equation:

(1) $$C=\frac{I\cdot \Delta t}{m\cdot \Delta V}$$

where *I* (A) is the charge/discharge current, *m* (g) is the mass of the active materials in the electrode, Δ*t* is the discharge time, and Δ*V* (V) is the potential window.

### 2.6. Fabrication of Asymmetric Supercapacitor (ASC) Devices

The ASC was assembled with PANI/LDH as a positive electrode and activated carbon as a negative electrode. In addition, the BET surface area and pore size of AC are about 901.0 m^2^ g^−1^ and 1.9 nm, respectively. The AC negative electrode was prepared by mixing activated carbon, acetylene black and polytetrafluoroethylene (PTFE) with a mass ratio of 80:10:10, and the mixture was coated on carbon cloth. The PVA-KOH gel electrolyte was prepared by dissolving PVA (3.0 g) in 20 mL deionized water with stirring at 85 °C for 4 h, then 10 mL KOH (2.0 g) solution was added into the PVA solution dropwise. The whole mixture was kept at 85 °C until the solution became clear. The PVA-KOH gel was then used for the electrolyte and separator of the asymmetric supercapacitor. The mass loading of active materials on negative and positive electrodes is evaluated according to the following equation to maintain a charge balance *q_+_* = *q_-_*

(2) $$\frac{m_{+}}{m_{-}}=\frac{C_{-}\times \Delta V_{-}}{C_{+}\times \Delta V_{+}}$$

where ΔV is the potential window and *C* is the mass specific capacitance of each electrode measured in three-electrode configuration. To balance the charges of the negative and positive electrode, the mass ratio is calculated to be $m_{+}$/$m_{-}\approx$ 0.255.

The energy density (E) and power density (P) based on GCD test were calculated by using the following equations:

(3) $$E=\frac{1}{2}CV^{2}$$

(4) $$P=\frac{E}{t}$$

where *E* (Wh kg^−1^), *C* (F g^−1^), *V* (V), *P* (W kg^−1^), and *t* (s) correspond to the energy density, specific capacitance, potential range, power density, and discharge time, respectively.

## 3. Results and Discussion

**Figure 1:**
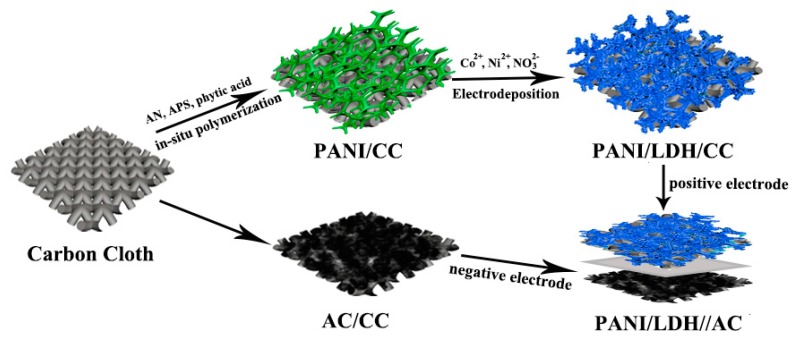
Schematic illustration for the preparation process of PANI/LDH/CC core-shell nanofibers and asymmetric supercapacitor.

The preparation process of core-shell PANI/LDH/CC composite nanofiber is schematically shown in Figure 1. PANI nanofibers are firstly formed on CC via in-situ oxidative polymerization with the aid of phytic acid, which then acts as a 3D conductive framework for growth of LDH nanosheets by electrodeposition. The resulting hierarchical architectures may facilitate mass transport and electron transfer and ensure the structural stability, giving rise to improved electrochemical properties of the PANI/LDH/CC electrode. Subsequently, an ASC device is assembled with PANI/LDH/CC as a positive electrode and AC/CC as a negative electrode in PVA-KOH gel electrolyte.

**Figure 2:**
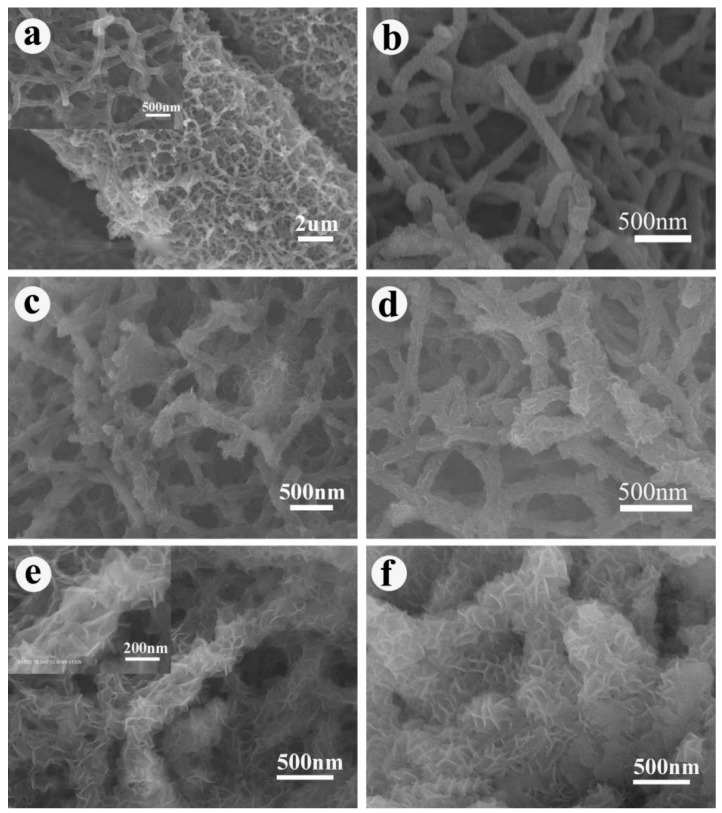
(**a**) FESEM micrograph of PANI network; (**b**–**f**) FESEM images of obtained PANI/LDH-50, PANI/LDH-100, PANI/LDH-150, PANI/LDH-200 and PANI/LDH-250, respectively; The inset in (**a**) and (**e**) shows high-magnification of respective FESEM images.

The morphologies of PANI/CC and PANI/LDH/CC were investigated by FESEM (Figure 2). As shown in Figure 2a, dense PANI nanofibers are uniformly anchored on the surface of CC. These fibers are curled and interconnected to form 3D porous network structure due to the doping and crosslinking effect of phytic acid [30]. Moreover, the magnified SEM image (inset of Figure 2a) clearly demonstrates that the PANI nanofibers are around 80–150 nm in diameter and 1–2 µm in length. After subsequent electrodeposition of LDH, the surface morphology of PANI varies with deposition time, as illustrated in Figure 2b–f. Apparently, compared with that of pristine PANI, the surface of PANI nanofibers becomes rough due to the growth of LDH (Figure 2b). After electrodeposition for 50 to 150 s, a growing number of LDH nanoflakes on the PANI skeleton can be observed distinctly and porous network architectures still remain (Figure 2c–d). As the deposition time is prolonged to 200 s, well-defined hierarchical core-shell nanostructures are formed with interlaced LDH nanoflakes coated on a PANI core, as revealed in Figure 2e. The enlarged SEM image in Figure 2e indicates that these ultrathin nanoflakes have an edge width of ~100 nm and thickness of ~10 nm. Hierarchical PANI/LDH composite with porous architectures not only provides a large contact area between the electrode and electrolyte but also facilitates ion and electron transfer. Simultaneously, the crosslinked networks ensure the structural stability and mechanical integrity of a composite electrode during charge–discharge processes. With the further increase of deposition time, the thickness of LDH nanosheets shell increases significantly and the voids between PANI/LDH composite nanofibers are thus blocked by the agglomeration of LDH nanosheets (Figure 2f), which undoubtedly decrease the active surface area for electrochemical reaction.

**Figure 3:**
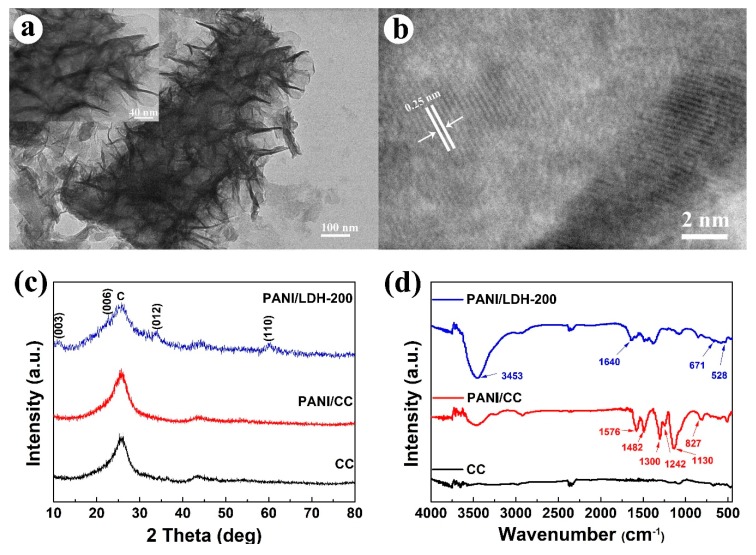
(**a**) TEM and (**b**) HRTEM image of PANI/LDH-200; the inset of (**a**) shows high-magnification of TEM image; (**c**) XRD patterns and (**d**) FTIR spectra of CC, PANI/CC and PANI/LDH-200.

The hierarchical core-shell structure is also verified by TEM image, as plotted in Figure 3a. Plenty of NiCo-LDH nanosheets spread evenly on PANI nanofibers, constructing typical hierarchical architecture with LDH as the shell and PANI as the core. High-magnification TEM of Figure 3a shows that the width of the core-shell nanorod increases up to approximately 240 nm after electrochemical deposition of NiCo-LDH for 200 s, which provides strong support for the SEM results. Moreover, as can be seen in Figure 3b, the lattice fringes of PANi/NiCo-LDH-200 from the high resolution TEM (HRTEM) display characteristic interplanar spacings of about 0.25 nm, which is in good agreement with the (012) lattice planes of NiCo-LDH [31]. 

Figure 3c depicts XRD patterns of CC, PANI/CC and PANI/LDH-200. Two broad peaks at 25.4° and 43.2° correspond to the characteristic peaks of carbon fiber [32]. It should be pointed out that the pattern of PANI/CC is similar to that of CC, implying that the obtained PANI is amorphous. After coating the NiCo-LDH nanosheet, the diffraction pattern for the PANI/LDH/CC shows four new broad peaks at 11.4°, 22.8°, 34.4°, and 60.7°, which are characteristic of (003), (006), (012), and (110) planes of NiCo-LDH [17,33]. The results indicate that the crystalline NiCo-LDH has grown on PANI chains successfully. In order to further investigate the structure of PANI/LDH composites, FTIR spectroscopy is performed. Figure 3d illustrates the FTIR of CC, PANI/CC and PANI/LDH/CC samples. Compared to the curve of CC, the spectrum of PANI/CC clearly shows the characteristic bands of PANI. The peaks at 1576 and 1482 cm^−1^ could be ascribed to the C=C stretching of quinoid (Q) and benzenoid (B) rings. The peaks at 1300 and 1242 cm^−1^ are caused by the C=N and C–N stretching, respectively. In addition, the peaks at 1130 and 827 cm^−1^ are associated with the stretching of N=Q=N and bending vibration of C–H [26,34]. These characteristic peaks demonstrate the successful polymerization of the PANI on CC. Upon electrodeposition of LDH nanosheets on PANI skeletons, the intensity of the characteristic peaks of PANI decreased dramatically, and some new peaks appeared in the spectrum of PANI/LDH-200. A broad peak observed at 3453 cm^−1^ is attributed to the stretching vibration of hydrogen-bonded hydroxyl groups on the Ni-Co LDH nanosheet. The peak at 1644 cm^−1^ is assigned to the bending vibration for the absorbed water molecule onto the NiCo-LDH [19]. The weak signals from 671 and 528 cm^−1^ are due to the stretching vibrations of Ni-O and Co-O, respectively [35,36].

**Figure 4:**
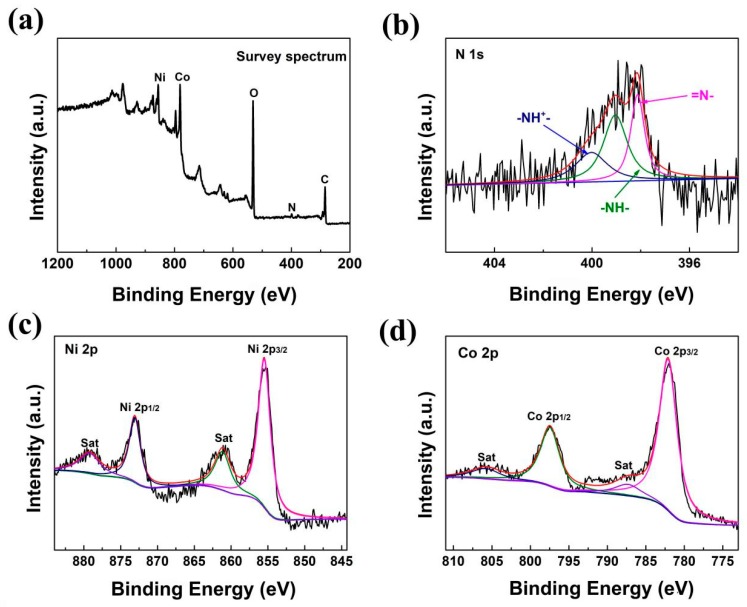
(**a**) XPS survey scan spectrum of PANI/LDH-200; (**b**) high-resolution N1s, (**c**)Ni 2p and (**d**) Co 2p spectra of PANI/LDH-200.

Further surface analysis of the PANI/LDH-200 is carried out by X-ray photoelectron spectroscopy. Figure 4a shows the overall XPS survey spectrum, which confirms the presence of C, N, O, Co, and Ni elements within the resultant PANI/LDH-200 composite. As shown in Figure 4b, the signal of N 1s can be split into three peaks at 400.1, 399.0 and 398.1 eV corresponding to the positively nitrogen cationic radical (-NH^+^-), the benzenoid amine nitrogen (-NH-) and the imine nitrogen (=N-), respectively [37]. The results indicate the existence of PANI. For the Ni 2p spectrum (Figure 4c), besides two shake-up satellites (denoted as “sat”), there is a pair of peaks located at 855.5 and 873.1 eV assigned to Ni 2p_3/2_ ans Ni 2p_1/2_, respectively, and the spin-energy separation of 17.6 eV between Ni 2p_3/2_ and Ni 2p_1/2_ reveals the presence of Ni^2+^ in Ni(OH)_2_ [19]. As shown in Figure 4d, the binding energy values centered at 782.2 and 797.5 eV correspond to Co 2p_1/2_ and Co 2p_3/2_, which affirms the existence of the Co^2+^ valence state [18,19]. Thus, the XPS analysis suggests that the NiCo-LDH shell is deposited successfully on the surface of PANI.

The nitrogen (N_2_) adsorption–desorption measurements were conducted to investigate the specific surface area and pore volume of the as-prepared PANI/LDH-T composites. As shown in Figure S1, the profiles of the hysteresis loop demonstrate adsorption–desorption characteristics of mesoporous materials. As evidenced by Table S1, the PANI/LDH-200 exhibits the highest specific surface area (138.4 m^2^ g^−1^) and largest pore volume (0.149 cm^3^ g^−1^), which may result in its superior electrochemical behavior to other composite electrodes.

**Figure 5:**
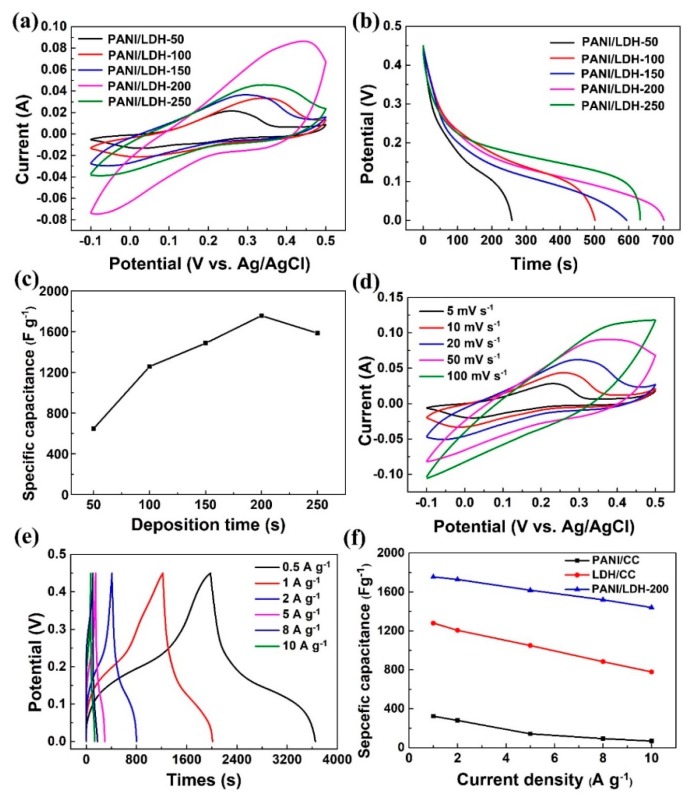
(**a**) CV curves of PANI/LDH-T (T = 50, 100, 150, 200, 250 s) at a scan rate of 20 mV s^−1^; (**b**) GCD curves of the PANI/LDH-T at a current density of 1.0 A g^−1^; (**c**) specific capacitance of different deposition times at a current density of 1.0 A g^−1^; (**d**) CV curves of the PANI/LDH-200 at various scan rates; (**e**) GCD curves of the PANI/LDH-200 at different current densities; (**f**) the rate capability of the PANI/CC, LDH /CC and PANI/LDH-200.

In order to evaluate the electrochemical properties of PANI/LDH with different electrodeposition times, a series of electrochemical tests are carried out in 2.0 M KOH solution with a three-electrode measurement system. Figure 5a shows the CV profiles of PANI/LDH composites with different deposition times at the scan rate of 20 mV s^−1^. All the curves exhibit visible reduction/oxidation peaks with a potential window ranging from −0.1 to 0.5 V, indicating faradaic reactions in the charge/discharge process owing to the reversible redox of X^2+^/X^3+^ (X = Co and Ni) [36]. It is worth noting that PANI/LDH-200 possesses the largest enclosed area of CV curves and highest peak current in contrast with the other electrodes, suggesting that 200 s of LDH electrodeposition can result in the largest specific capacitance of the PANI/LDH composite. However, with further prolongation of the deposition time, the integral area of the CV curve for PANI/LDH-250 decreases dramatically, in addition, its corresponding capacitance drops as well because of the redundant deposition of NiCo-LDH nanosheets. Figure 5b displays galvanostatic discharge curves of PANI/LDH composites at a current density of 1.0 A g^−1^. It is evident that PANI/LDH-200 electrode demonstrates the longest discharge time, indicating the largest specific capacitance. These trends in capacitance of composite electrodes are also in line with the CV results. Based on Equation (1) and the discharge curves, the calculated specific capacitance as a function of the current density is shown in Figure 5c. The capacitance of PANI/LDH-200 reaches 1755 F g^−1^ at 1.0 A g^−1^, whereas those of other PANI/LDH-T (T = 50, 100, 150, 250 s) electrodes are 647, 1257, 1485, and 1584 F g^−1^, respectively. The significantly improved capacitance of the PANI/LDH-200 composite could be ascribed to its unique hierarchical core-shell structure with a porous crosslinked network. On one hand, highly conductive PANI nanofibers on CC serve both as the skeleton and transport channel for charge storage and delivery, and thus enhance the conductivity of PANI/NiCo-LDH composite electrode. On the other hand, ultrathin NiCO-LDH nanosheets on crosslinked PANI networks not only enlarge the contact area between electrolyte and PANI/NiCo-LDH electrode, but also facilitate ion diffusion into the inner region of the electrode to maximize the utilization of LDH and PANI pseudocapacitive materials. In addition, the void spaces between the interlaced LDH nanosheets are also beneficial to electrolyte penetration as well as to buffer the strain change of an electrode during redox reaction.

Since PANI/LDH-200 composite exhibits the optimum capacity, its electrochemical properties are thus investigated in the following discussion. Figure 5d shows the CV curves of the PANI/LDH-200 composite electrode at various scan rates from 5–100 mV s^−1^. Typically, a pair of redox peaks at the scan rate below 50 mV s^−1^ can be clearly observed, demonstrating the battery-type behavior of the PANI/LDH-200 electrode [38]. Along with the rise of scan rate, the peak current approximately increases linearly, suggesting the fast ion and electron transport of the composite electrode. At a higher scan rate of 100 mV s^−1^, the redox peaks become weak due to insufficient redox reactions and the polarization effect of the electrode. To further quantify the electrochemical behavior of the PANI/LDH-200 electrode, its GCD curves at different current densities are plotted in Figure 5e. The nonlinear curves with charge–discharge platforms indicate the pseudocapacitive characteristics of the composite corresponding to the redox peaks observed in the CV profile. Meanwhile, all the nearly symmetric GCD curves reveal that the redox reactions between the PANI/LDH-200 electrode and KOH eletrolyte are highly reversible. Impressively, the PANI/LDH-200 delivers the largest capacitances of 1845 F g^−1^ at a current density of 0.5 A g^−1^, which is superior to those of other reported LDH-based composite electrodes [17,39,40,41,42,43] as shown in Table S2.

The rate performance of PANI/CC, LDH/CC and PANI/LDH-200/CC are also evaluated by GCD measurements. Figure 5f displays the specific capacitance of these samples as a function of the current density. Apparently, all the electrodes exhibit a gradual degradation in the capacitance with the current density due to reduced active surface area for charge storage, high ohmic drop and partial access of ions to the pores within the electrodes. More strikingly, the capacitance of PANI/LDH-200/CC is significantly larger than that of the PANI/CC and LDH/CC over the whole current density. With the current density increasing from 1.0 to 10.0 A g^−1^, the capacitance retention of the PANI/LDH-200/CC composite still comes up to 82.1%, much higher than that of PANI/CC (22.1%) and LDH/CC (60.8%), respectively, which definitely demonstrates the superior rate capability of the PANI/LDH-200/CC ternary composite electrode. Core-shell PANI/LDH composite with ultrathin LDH nanoflakes on porous PANI networks makes the electrolyte solution have easy permeation into inner active sites of the electrode and shortens the distance of OH- diffusion. On the other hand, the intersecting LDH nanoflakes not only help alleviate the strain from the high rate of insertion/extraction of ions but also accommodates the volume change of PANI, leading to adequate redox reactions even at a high current density. As a result, the PANI/LDH/CC composite possesses better rate performance than the other two binary ones.

**Figure 6:**
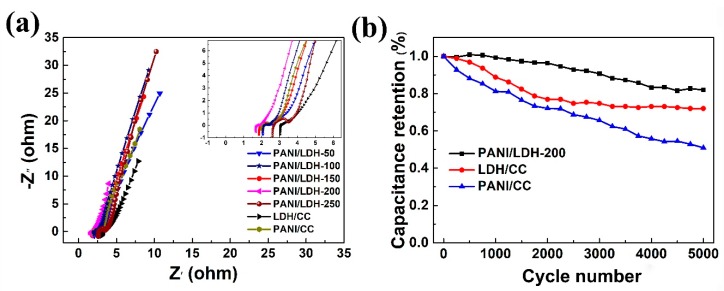
(**a**) Nyquist plots of LDH/CC, PANI/CC and PANI/LDH-T (T = 50, 100, 150, 200, 250), the inset indicates the enlarged Nyquist plots at high-frequency region; (**b**) cycling stability of the LDH/CC, PANI/CC and PANI/LDH-200.

Electrochemical impedance spectroscopy (EIS) is also conducted to analyze the electrochemical behavior of the obtained composites. Figure 6a shows the corresponding Nyquist plots of PANI/CC, LDH/CC and PANI/LDH-T; all the curves contain a semicircle in the high frequency region and a straight line in the low frequency region, which demonstrates the capacitive behavior of the electrode materials. In the high frequency region, the intercept at the real axis is related to the equivalent series resistance (ESR). As indicated by the spectrum, the ESR value of PANI/LDH-200 is smaller than that of other electrodes, which reflects the lower internal resistance and faster electron transfer kinetics of the PANI/LDH-200 electrode. The diameter of the semicircle represents the charge transfer resistance (Rct) at the electrode–electrolyte interface [44]. Obviously, the PANI/LDH-200 exhibits much smaller Rct than the other electrodes, illustrating that the combination of LDH nanosheets with PANI nanofiber networks effectively enlarges the ion-accessible surface area and enables rapid ion transport into the inner region of the electrode. In addition, in the low frequency region, the larger slope of the straight line for PANI/LDH-200 electrode reveals the faster ions’ diffusion into the active materials. Therefore, the above results further confirm the superior electrochemical properties of the PANI/LDH-200 composite.

Electrochemical stability of PANI/CC, LDH/CC and PANI/LDH-200 composite electrodes are measured under a GCD test at 10.0 A g^−1^ for 5000 cycles. As shown in Figure 6b, the capacitance of PANI/LDH-200 almost remains unchanged until the end of 800 cycles and then declines, but still retains 82.0% of its initial capacitance after 5000 cycles at such high current density, suggesting its excellent cycling stability, while a gradual drop of capacitance for LDH/CC and PANI/CC composites can be observed and their capacitance retention is 72.0% and 50.9%, respectively. The low cycling stability of PANI is caused by the intrinsic volumetric swelling and shrinking during the charge–discharge process. However, after coating LDH nanosheets on PANI core to form a 3D hierarchical core-shell structure, the outer LDH shell effectively relieves the volume effect of PANI. Likewise, the crosslinked networks also endow the composite with robust architecture to prevent the structural breakdown during long-term redox reactions, thereby resulting in the significantly enhanced cycling stability of the PANI/LDH-200 composite electrode. 

**Figure 7:**
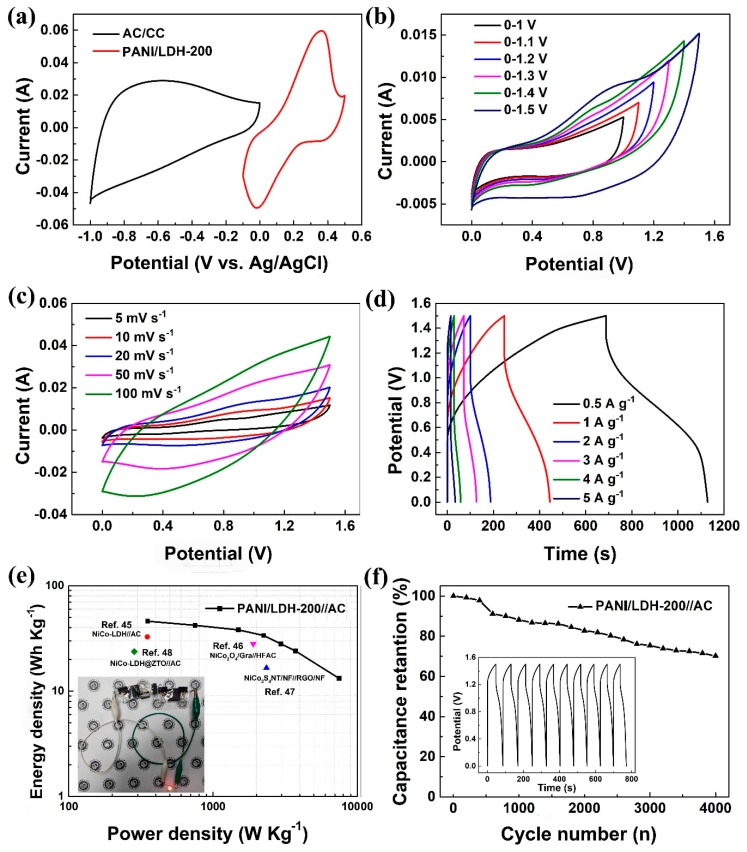
(**a**) CV curves of AC and PANI/LDH-200 at a scan rate of 20 mV s^−1^; (**b**) CV curves at a scan rate of 20 mV s^−1^ within different potential windows; (**c**) CV curves of the PANI/LDH-200//AC at various scan rates in a potential window of 0 to 1.5 V; (**d**) GCD curves of the PANI/LDH-200//AC at different current densities; (**e**) Ragone plot of the ASC, inset shows a photo of the ASC device; (**f**) cycling stability of the ASC at a scan rate of 4.0 A g^−1^, and the inset shows the GCD curves of the last 10 cycles.

In view of the excellent performance of the PANI/LDH electrode, in order to investigate its practical applications, an asymmetric supercapacitor with PANI/LDH-200 as the positive electrode and AC as the negative electrode in PVA-KOH electrolyte is assembled. The positive electrode of PANI/LDH-200 possesses a high pseudocapacitance, which is helpful for increasing the energy density of the asymmetric supercapacitor, while the activated carbon (AC) negative electrode with rich pore size and large specific surface area is beneficial to the rapid insertion and extraction of hydroxide, in favor of improving the power density of the device. Figure 7a shows the CV curves of the AC/CC and PANI/LDH-200 electrodes measured at a scan rate of 20 mV s^−1^ in a three-electrode system. Based on the principle of charge balance, the mass loading of AC and PANI/LDH-200 is around 9.8 and 2.5 mg cm^−2^, respectively. It is clear that the AC/CC electrode displays a typical electric double-layer capacitor behavior in the potential range of −1.0 to 0 V, whereas the PANI/LDH-200 electrode shows a pseudocapacitive characteristic with a potential window from −0.1 to 0.5 V. In order to obtain the optimal operating voltage of a PANI/LDH-200//AC device, a series of CV curves under various potential windows are recorded at 20 mV s^−1^ (Figure 7b). The potential window can be extended to 1.5 V where the curve does not show obvious polarization. Figure 7c illustrates the CV curves of the ASC within a potential window of 1.5 V at different scan rates. Quasi-rectangular CV curves indicate the ideal capacitive behavior of the assembled device. Weak redox peaks at scan rates from 5 to 100 mV s^−1^ confirm the combined contribution from electric double-layer capacitance (EDLC) and pseudocapacitance. Furthermore, the shape of the CV curve is not distorted obviously even at the high sweep rate of 100 mV s^−1^, demonstrating a fast response rate and good rate capability of PANI/LDH//AC. 

Figure 7d shows the GCD curves of PANI/LDH-200//AC at different current densities from 0.5 to 10 A g^−1^. Based on the GCD curves, the calculated specific capacitance of the device reaches to 147.2 F g^−1^ at 0.5 A g^−1^. Even at a high current density of 5.0 A g^−1^, it still remains 70.8 F g^−1^. At the same time, the GCD curves are used to calculate the energy density and power density at different current densities. Figure 7e depicts the Ragone plot of the fabricated PANI/LDH-200//AC. At a current density of 0.5 A g^−1^, the ASC achieved a maximum energy density of 46.0 Wh kg^−1^ at a power density of 351.6 W kg^−1^. Even at a power density of 7.5 kW kg^−1^ (10 A g^−1^), the energy density is still 13.16 Wh kg^−1^, which is higher than previously reported ASC devices such as Ni-Co LDH//AC (32.67 Wh kg^−1^ at 350.0 W kg^−1^) [45], NiCo_2_O_4_ /Gra//HFAC (28.0 Wh kg^−1^ at 1.9 kW kg^−1^) [46], NiCo_2_S_4_ NT/NF//PGO/NF (16.6 Wh kg^−1^ at 2.35 kW kg^−1^) [47] and Ni-Co LDH@ZTO//AC (23.7 Wh kg^−1^ at 284.2 W kg^−1^) [48]. For the purpose of application, the cyclic stability of the PANI/LDH-200//AC is also tested at a current density of 4.0 A g^−1^ for 4000 cycles (Figure 7f). The ASC device shows a capacitance retention of 70.2%, and the Coulombic efficiency calculated from GCD curves for the last 10 cycles (Figure 7f, inset) is around 95.6%, suggesting good cycling performance of the device. In order to highlight its potential application, a red light-emitting diode (LED) is lit up by two charged devices connected in series (inset of Figure 7e), demonstrating its great application potential for energy storage systems.

## 4. Conclusions

In summary, PANI/LDH hierarchical core-shell networks are successfully synthesized by in-situ polymerization and the electrodeposition method. The 3D networks of PANI are beneficial to the growth of NiCo-LDH nanosheets and shorten electrons and ions transfer distance. Meanwhile, the hierarchical core-shell nanostructure improves the contact area between electrolyte ions and active materials. The LDH nanosheets shell growing on the PANI network not only facilitates ion and electron transport but also relieves the strain change of the electrode during redox reaction. Hence, the well-defined PANI/LDH composite electrode delivers a high mass specific capacitance of 1845 F g^−1^ at 0.5 A g^−1^ and outstanding rate capability with a capacitance retention of 82.1% from 1.0 to 10 A g^−1^. The ASC device shows an excellent energy density of 46.0 Wh kg^−1^ at a power density of 351.6 W kg^−1^ and good cycling performance. The results highlight attractive applications of PANI/LDH electrodes in energy storage devices.

## Supplementary Materials

The following are available online at https://www.mdpi.com/2079-4991/9/4/527/s1, Figure S1: N_2_ adsorption/desorption isotherms of PANI/LDH-T composites, Table S1: The surface area and pore volume of all samples, Table S2: Comparison of the electrochemical performance of the PANI/LDH electrode with those in previous reports.
