# Supplementary material for: Hierarchical PANI/NiCo-LDH Core-Shell Composite Networks on Carbon Cloth for High Performance Asymmetric Supercapacitor
Source: Nanomaterials (Basel). 2019 Apr 3;9(4):527. doi: 10.3390/nano9040527 (PMC6523088; doi:10.3390/nano9040527)
Supplement: Supplementary file 1 [file nanomaterials-09-00527-s001.pdf]

Supplementary Material

Hierarchical PANI/NiCo-LDH Core-shell Composite Networks on Carbon Cloth for High Performance Asymmetric Supercapacitor

Xinjin Ge [1], Ying He [1,2,*], Tomas Plachy [2], Natalia Kazantseva [2], Petr Saha [2] and Qilin Cheng [1,2,*]

[1] Key Laboratory for Ultrafine Materials of Ministry of Education, School of Materials Science and Engineering, East China University of Science and Technology, Shanghai 200237, China; 18818217542@163.com (X.G.)

[2] Centre of Polymer Systems, Tomas Bata University in Zlin, nam. Masaryka T.G. 5555, 760 01 Zlin, Czech Republic; plachy@utb.cz (T.P.); kazantseva@utb.cz (N.K.); saha@utb.cz (P.S.)

* Correspondence: rehey@ecust.edu.cn (Y.H.); chengql@ecust.edu.cn (Q.C.)

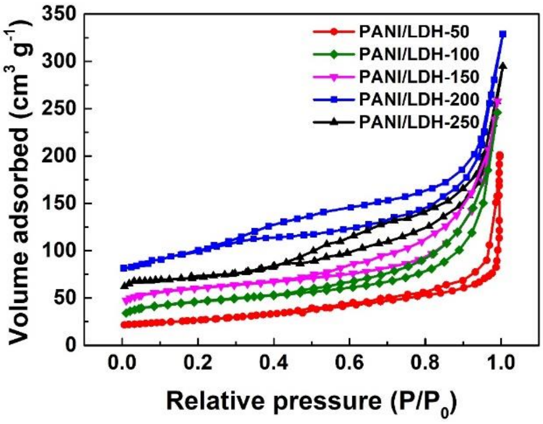

Figure S1. N_2 adsorption/desorption isotherms of PANI/LDH-T composites.

Table S1. The surface area and pore volume of all samples.

Composite Electrode	S_{BET} (m^2 g^{-1})	Pore Volume (cm^3 g^{-1})
PANI/LDH-50	70.9	0.068
PANI/LDH100	92.7	0.086
PANI/LDH-150	108.5	0.113
PANI/LDH-200	138.4	0.149
PANI/LDH-250	114.3	0.125

Table S2. Comparison of the electrochemical performance of the PANI/LDH electrode with those in previous reports.

Electrode structure	Specific capacitance (F g^{-1})	Capacitance retention (%)	Reference
$Ni_{50}Co_{50}$-LDH	1537 F g^{-1} at 0.5 A g^{-1}	80.3% (1000)	17
PANC@Co–Ni LDH	1529.52 F g^{-1} at 0.5 A g^{-1}	/	39
LDH–CNT/RGO	1188 F g^{-1} at 1 A g^{-1}	>100% (1000)	40
Ni–Co LDH	1265 F g^{-1} at 1Ag^{-1}	92.9% (2000)	41
MXene-LDH	983.6 F g^{-1} at 2 A g^{-1}	76% (5000)	42
PMNC/G-2	1809 F g^{-1} at 0.5 A g^{-1}	83% (10000)	43
PANI/LDH	1845 F g^{-1} at 0.5 A g^{-1}	82% (5000)	This work
